# Population health implications of exposure to pervasive military aircraft noise pollution

**DOI:** 10.1038/s41370-024-00670-1

**Published:** 2024-05-09

**Authors:** Giordano Jacuzzi, Lauren M. Kuehne, Anne Harvey, Christine Hurley, Robert Wilbur, Edmund Seto, Julian D. Olden

**Affiliations:** 1 School of Aquatic and Fishery Sciences, College of the Environment, University of Washington, Seattle, WA 98195 USA; 2 Omfishient Consulting, Bremerton, WA 98310 USA; 3 Sound Defense Alliance, PO Box 373, Coupeville, WA 98239 USA; 4 Citizens of Ebey’s Reserve, PO Box 202, Coupeville, WA 98239 USA; 5 School of Public Health, University of Washington, Seattle, WA 98195 USA

**Keywords:** Noise assessment, Environmental noise, Exposure-response, Dasymetric, Annoyance, Sleep disturbance

## Abstract

**Background:**

While the adverse health effects of civil aircraft noise are relatively well studied, impacts associated with more intense and intermittent noise from military aviation have been rarely assessed. In recent years, increased training at Naval Air Station Whidbey Island, USA has raised concerns regarding the public health and well-being implications of noise from military aviation.

**Objective:**

This study assessed the public health risks of military aircraft noise by developing a systematic workflow that uses acoustic and aircraft operations data to map noise exposure and predict health outcomes at the population scale.

**Methods:**

Acoustic data encompassing seven years of monitoring efforts were integrated with flight operations data for 2020–2021 and a Department of Defense noise simulation model to characterize the noise regime. The model produced contours for day-night, nighttime, and 24-h average levels, which were validated by field monitoring and mapped to yield the estimated noise burden. Established thresholds and exposure-response relationships were used to predict the population subject to potential noise-related health effects, including annoyance, sleep disturbance, hearing impairment, and delays in childhood learning.

**Results:**

Over 74,000 people within the area of aircraft noise exposure were at risk of adverse health effects. Of those exposed, substantial numbers were estimated to be highly annoyed and highly sleep disturbed, and several schools were exposed to levels that place them at risk of delay in childhood learning. Noise in some areas exceeded thresholds established by federal regulations for public health, residential land use and noise mitigation action, as well as the ranges of established exposure-response relationships.

**Impact statement:**

This study quantified the extensive spatial scale and population health burden of noise from military aviation. We employed a novel GIS-based workflow for relating mapped distributions of aircraft noise exposure to a suite of public health outcomes by integrating acoustic monitoring and simulation data with a dasymetric population density map. This approach enables the evaluation of population health impacts due to past, current, and future proposed military operations. Moreover, it can be modified for application to other environmental noise sources and offers an improved open-source tool to assess the population health implications of environmental noise exposure, inform at-risk communities, and guide efforts in noise mitigation and policy governing noise legislation, urban planning, and land use.

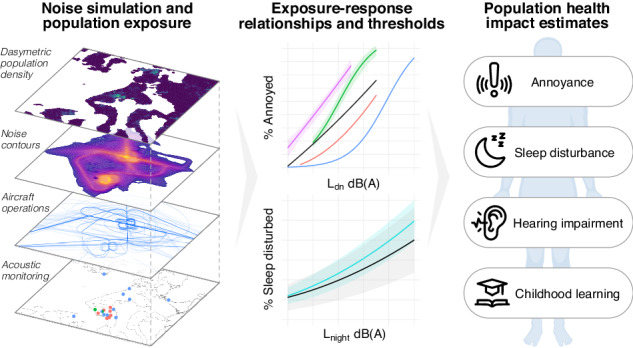

## Introduction

The adverse effects of noise pollution, or unwanted or disturbing sound, on human physical and psychological health are widely recognized. Documented impacts range from annoyance and stress to elevated risk of sleep disturbance, cardiovascular disease, hearing impairment, and compromised childhood learning [1–5]. Recognition of these impacts and their associated costs to public health have highlighted the problem of increasing environmental noise originating from diverse human activities [6, 7]. Of these, noise from pervasive aviation is a primary and growing cause of concern internationally and has been reported to elicit more severe health impacts than other sources of transportation noise [8–10].

The ability to assess the health consequences of aircraft noise is fundamental to inform affected communities about risks, devise strategies to mitigate impacts, and guide policy to protect or restore public health. In recent decades, growing scientific inquiry has led to the development of quantitative exposure-response relationships that directly link aircraft noise exposure with estimated population health outcomes [9, 11, 12]. However, while policies enacted in a handful of regions (e.g., European Noise Directive, Environmental Quality Standards for Noise) have improved the assessment of environmental noise exposure and health impacts, regulatory actions to reduce noise pollution and/or mitigate exposures have largely been slow and irregular [13–16]. Furthermore, compared to research in civil aviation noise (i.e., commercial and private aircraft), studies concerned with the health implications of noise from military aviation specifically have faced unique challenges (e.g., intermittent or unpublished operations, a lack of data to inform exposure-response relationships) that have limited the scale and scope of data available to assess population health risks [17–19].

Noise regimes of military aviation differ dramatically in their level, spectra, repetition, and character [17, 18, 20–23]. Unlike commercial aircraft noise, which is consistent and largely predictable in the frequency of events and duration of exposure, noise from military activity can vary widely over time. Military air bases are ubiquitous around the world, and a small but growing body of research has begun to investigate the unique human health implications of military aircraft noise exposure. For example, noise from military aviation can elicit different, and often greater, impacts on human disturbance and health compared to civil aviation [19, 24, 25]. These studies remain in the minority, leading to insufficient understanding to inform impacted communities and guide specialized policy [7]. Furthermore, military airfields and air spaces may often be exempted from or subject to differing regulations for noise assessment and mitigation [26, 27], leading to a policy or regulatory vacuum whereby communities must petition for such actions. For example, although federal policy allows compensation for sound insulation in high exposure areas around civilian airports in the US, this policy applies only to “public use airports” [27]. While the spatiotemporal extent of operations and noise exposure from American civilian transportation is readily available (e.g., US National Transportation Noise Map [28]), corresponding data on how military aircraft operations are the source of noise permeating across space and time is reported much less often to the public.

In recent years, public concern has grown regarding noise experienced by communities in Washington State, USA, from military training activities at Naval Air Station Whidbey Island (NASWI) [29]. Since the US Navy consolidated its fleet of EA-18G Growler aircraft at NASWI and expanded flight operations in 2013, noise exposures have grown dramatically for residents of multiple counties [17, 18]. An environmental impact statement was conducted in accordance with the National Environmental Protection Act, which provided evidence for community annoyance, speech and classroom interference, as well as an increase in the probability of awakening and the population vulnerable to potential hearing loss due to military aircraft noise events [30]. However, the assessment of population health impacts has consistently come under scrutiny, and as early as 2017 the Washington State Board of Health concluded that available data was insufficient to assess the impacts of a proposed operational increase, and a full public health risk assessment was needed [31]. This was supported by a subsequent review that found that operations around NASWI largely exceeded those of all health-related studies of military low-elevation flights worldwide [17]. A lawsuit jointly filed by a citizens group and Washington State in 2019 ultimately resulted in a 2022 ruling that the Navy did not adequately consider ramifications for childhood learning, and a subsequent order to reconduct the environmental impact statement [32].

The present study sought to address this knowledge gap by conducting a transparent and reproducible quantitative assessment of military aviation noise and its implications for public health and well-being at a regional scale. Employing a novel workflow for evaluating the human health impacts of noise pollution, we quantify the sonic character and spatiotemporal distribution of aircraft noise exposure by integrating acoustic monitoring and simulations of aircraft activity with a population density map to ultimately derive a suite of population health outcomes, including estimates of annoyance, sleep disturbance, hearing impairment, and compromised childhood learning throughout the study region. This research was guided by input from community partners, and public webinars reported routinely on progress and outcomes throughout the entire study period. Results from this study provide evidence for the pervasive noise pollution, and resulting public health implications, stemming from military aviation at NASWI. More broadly, it provides a workflow to systematically assess the population health risks of noise pollution from sources other than military aircraft, which could be used as a basis for future environmental and public health impact assessments.

## Materials and methods

### Study region

**Fig. 1:**
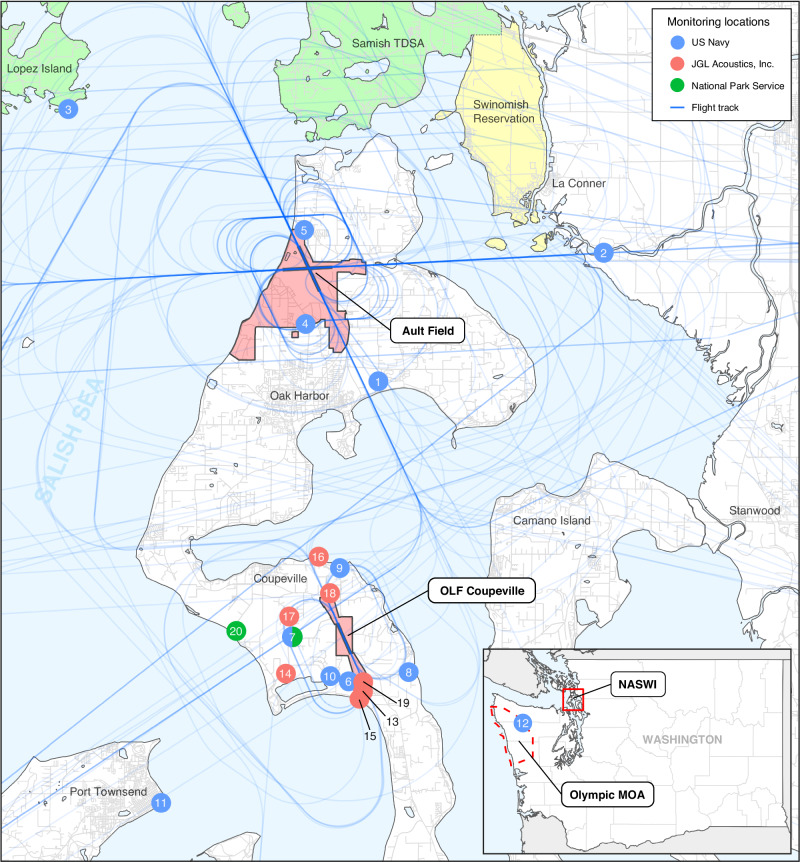
Map of the study region, including Ault Field and Outlying Landing Field (OLF) Coupeville of Naval Air Station Whidbey Island (NASWI) and the Olympic Military Operations Area (MOA). Monitoring locations are shown from the US Navy, JGL Acoustics Inc., and the National Park Service. The Swinomish Reservation and Samish Tribal Designated Statistical Area are indicated in yellow and green, respectively.

Military training operations at NASWI originate from two primary airfields on Whidbey Island, Washington State, USA (Fig. 1). Ault Field is located approximately 5 km from the city of Oak Harbor, the largest community in Island County, while Outlying Landing Field (OLF) Coupeville is located 4 km from the town of Coupeville. Aircraft operations conducted at NASWI range from sessions of repeated closed-pattern routines (including “touch-and-go” field carrier landing practice, FCLP), to interfacility transfers and arrivals from and departures to off-station areas, including the Olympic Military Operations Area (MOA) on the Olympic Peninsula (the primary location of electronic warfare and air-to-air combat training). The flight paths for these operations extend across northwestern Washington, from the Pacific coast to the Cascade Mountains, encompassing the counties of Clallam, Jefferson, Island, San Juan, Skagit, and Snohomish, in Washington State, USA.

### Analysis workflow

**Fig. 2:**
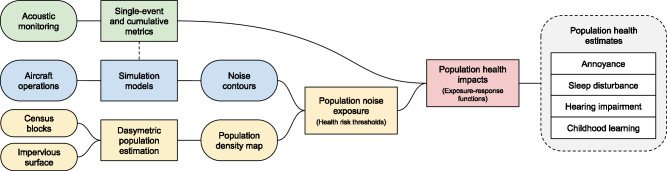
Diagram of analysis workflow. Data are shown as oblongs, while models and processing steps are shown as rectangles.

Acoustic metrics characterizing individual aircraft noise events and cumulative exposure levels were derived from acoustic data recorded at monitoring locations and used to validate a model simulating noise exposure across the entire study region. Modeled spatial predictions, expressed as noise contours, were overlaid with a dasymetric population density map to estimate population noise exposures at a fine spatial scale. Established thresholds and exposure-response functions were used to estimate the effect of the noise regime on multiple population health outcomes. This analysis workflow is detailed in Fig. 2.

### Acoustic monitoring data and metrics

Acoustic monitoring data consisted of sound pressure level (SPL) measurements collected during previous investigations into military aircraft noise, primarily from locations near the Ault and Coupeville airfields or their associated flight paths, both on Whidbey Island and throughout the surrounding region. Congress passed unique legislation in 2019 requiring the Navy to conduct acoustic monitoring around NASWI during four discrete weeks in 2020–2021, and at one location within the Olympic MOA for 365 days [33]. These data were obtained from the Naval Facilities Engineering Systems Command [34]. Additional monitoring data from 2015 to 2019 were obtained from JGL Acoustics, Inc. and the National Park Service Night Skies and Natural Sounds Division [21, 35, 36]. In total, 20 unique locations were examined (Supplementary Table S1). SPL measurements were conducted with a class 1 sound level meter at a 1 Hz sampling rate and included A frequency-weighted equivalent continuous SPL L_Aeq_ and, where available, A-weighted fast time-weighted maximum SPL L_AFmax_ and peak C-weighted L_Cpeak_. Frequency spectrum measurements consisted of Z-weighted L_Zeq_ in one-third octave bands and were only available for a subset of locations. Further details regarding data collection can be found in the relevant references [21, 35–37].

We calculated a suite of acoustic metrics to characterize noise from single overflight events and cumulative noise levels associated with aircraft operations. Metrics were selected for their ubiquity in domestic and international standards and policy for land use compatibility, and because they provide the basis for exposure-response relationships concerning human health impacts [7, 9, 11, 12, 38–40]. All metrics throughout this study use A frequency weighting unless otherwise specified.

Single event metrics included the sound exposure level L_E_ (also referred to as SEL), the 1-second average event maximum L_max_, the fast time-weighted maximum L_Fmax_, and (when available) the instantaneous C-weighted peak sound pressure level L_Cpeak_. All metrics were calculated in accordance with standards established by the International Organization for Standardization (ISO) and the Navy [11, 37]. The spectral content of noise events was measured in one-third octave frequency bands for a subset of monitoring locations near Coupeville airfield (locations 6-10) having a high prevalence of FCLP aircraft events. Spectrums were energy-averaged for individual events, then energy-averaged within sites to yield a representative FCLP for each location.

Overflight events were detected from continuous SPL time-series data according to guidelines established in ISO 20906 and the SAE Aerospace Recommended Practice [38, 41], and following the approach used by the Navy for noise monitoring [37]. A 10 second moving average was applied to each SPL time-series, smoothing the signal and reducing small variations that might otherwise be incorrectly labeled as events. An individual event was detected when this level exceeded a threshold varying with ambient conditions; ISO procedures recommend estimating background sound by the 95% exceedance level of total sound L_95_, and aircraft maxima should measure at least 15 dB above residual sound [38]. We note that some time-series data were collected only during periods of active aircraft operations (Supplementary Table S1) and lacked a representative reference background. The threshold for event detection for these time-series was the maximum value between the L_95_ of the hour (+/−30 min) and a baseline 35 dB + 15 = 50 dB ambient value for each second. An event was determined to terminate when the level fell and remained below the threshold for 5 s. Detected events containing multiple peaks above a local exceedance threshold (e.g., due to rapid flybys or multiple aircraft operating simultaneously) were subdivided into individual events corresponding with each peak. Detected acoustic events at locations 1–12 were cross-referenced against reported events from the Navy [34] and verified as military aircraft events accordingly. Detected events at locations 13–20 were manually verified by a trained observer [21, 35, 36].

Cumulative metrics quantify noise exposure over periods of time and form the basis of most community or public health impact assessments. Calculated cumulative metrics included: L_dn_, the day-night average sound level (also referred to as DNL), with a +10 dB penalty applied to nighttime periods (22:00-07:00); L_den_, the day-evening-night average sound level, with a penalty of +5 and +10 dB applied to evening (19:00-22:00) and nighttime (22:00-07:00) periods, respectively; L_night_, the equivalent continuous sound pressure level during nighttime hours; and L_eqH_, the equivalent continuous sound pressure level over a specified time period *H*, such as 24 h. Cumulative noise exposure within the Olympic MOA was quantified only with L_dnmr_, the onset-rate adjusted monthly day-night average sound level, as it is conventionally used to account for the sporadic nature and potentially high onset rates of noise within special-use airspace [37].

Cumulative acoustic metrics were calculated for every monitoring location and date, including L_dn_, L_den_, L_night_, L_eq24h_, and hourly L_eq_. These metrics were computed directly from continuous time-series measurements L_eq,1s_, rather than an aggregation of individual noise events L_E_, in accordance with ISO standards [11] and to enable direct comparisons of ambient noise levels on days with and without flight operations.

### Aircraft operations data and simulation models

Detailed flight operations records were obtained from the Naval Facilities Engineering Systems Command for the four weeklong monitoring periods in 2020 and 2021, which were designed to capture “a range of flight operations across a range of seasonal weather conditions… during periods of high, medium, and low flight activity” [34, 37]. These records documented flight profile and track activity from Ault Field and OLF Coupeville, as well as maintenance and engine run-up operations. Records for training routes and airspace profiles within the Olympic MOA were also obtained for a 365-day period within 2020 and 2021. These data were originally collected for the Navy Real-Time Aircraft Sound Monitoring Study [33] and presented a unique opportunity to investigate direct links between military aircraft operations and the noise regime.

We used the Noisemap software suite to simulate and spatially map noise exposure across the study region [42]. Noisemap is a noise modeling tool approved by the United States Department of Defense and used by the Navy to predict noise from flight operations. It integrates airfield operational data, flight profile specifications (including track, altitude, and speed), and a library of reference noise measurements with environmental terrain data to simulate the acoustic propagation of generated noise and resulting exposure at a grid of points on the ground level. The number of operations used by Noisemap is based on the average annual day, or the average number of airfield operations that would occur during a single day assuming 365 days of flying per year [37]. The average number of total operations during the four discrete monitoring periods was approximately 83% of the projected total operations for an “average year” at NASWI for 2021 [30], thus underestimating true flight activity at the annual scale.

Operations data were summarized as the total number of operations per flight profile for each period, and the mean number of operations per flight profile was calculated across all monitoring periods. This yielded a final model representing average flight activity across all periods throughout the year. Noisemap then simulated this activity, including additional noise due to maintenance and preflight ground run-up operations, such that the total predicted aircraft noise exposure was the accumulated noise exposure generated from all active operations of aircraft on all flight profiles [42].

The Noisemap model produced noise exposure contours in 1 dB increments for L_dn_, L_night_, and L_eq24h_ from a grid of points spaced evenly at a standard distance of 914 m, or 3000 ft. The model also calculated noise exposure at specific locations corresponding to monitoring locations 1–11 to enable comparison of simulated noise metrics with those empirically measured by acoustic monitoring in the field. A second simulation was created to estimate noise exposure within the Olympic MOA using operations data averaged across the year.

Lastly, we applied the models to simulate the health impacts of alternative noise regimes by scaling the relative quantity of total flight operations across the range of 50–150%, ultimately projecting the response of population health outcomes to decreases or increases in aircraft activity. While this included estimates for the total number of operations projected for 2021 from the Navy environmental impact statement, it should be noted that this simple scaling of operations quantities from the four discrete monitoring periods does not accurately reflect the true operations and fleet composition active throughout 2021, and the projected population impact estimates are not representative, but rather demonstrative.

### Population noise exposure

US population distributions are often derived from census units, which vary in geographic size based on population density. Units in urban areas are typically small with evenly distributed populations, while units in rural areas are larger with irregularly distributed populations. Using census units as a basis for population assessment can substantially limit the resolution of any spatial analysis of rural communities, and can reduce the accuracy of estimated impacts from socio-environmental problems [43, 44].

To overcome this limitation, we implemented a workflow established by Swanwick et al. to create a 30-m resolution population density estimate for the study area [45]. This approach dasymetrically distributed block-level population estimates across all non-transportation impervious surfaces for each census block in the study area. We used the same approach to estimate population density for federally- and state-recognized tribal reservations and tribal-designated statistical areas (TDSA). Population data were obtained from the US Census Bureau’s 2021 American Community Survey, and impervious surface area data from the most recently available 2019 National Land Cover Database [46]. Simulated noise contours produced from Noisemap were rasterized to the same 30-m resolution as the population density map and intersected to yield an estimate of the number of people exposed to noise levels at or above thresholds established by domestic policy and international guidelines and associated with a substantial risk of impact on human health.

The World Health Organization (WHO) strongly recommends reducing aircraft noise levels below 45 dB L_den_, as aircraft noise above this level is associated with adverse health effects [9]. As such, we considered the 45 dB L_dn_ contour to represent the spatial extent of adverse cumulative noise exposure, and the population residing within this area was therefore exposed to quantities of noise known to be harmful to human health. Additional thresholds used to estimate the at-risk population included aircraft noise levels associated with annoyance (45 dB L_den_) [9], adverse effects on sleep (40 dB L_night_) [9], a risk of hearing impairment over time (70 dBA L_eq24_) [3, 39], and land use incompatibility according to regulations set by the Navy, Federal Aviation Administration (FAA), and US Department of Housing and Urban Development (65 dB L_dn_) [27, 47, 48]. The number of individuals predicted to be impacted by these health risks vary according to the relationships described in the following section.

### Population health impacts

**Fig. 3:**
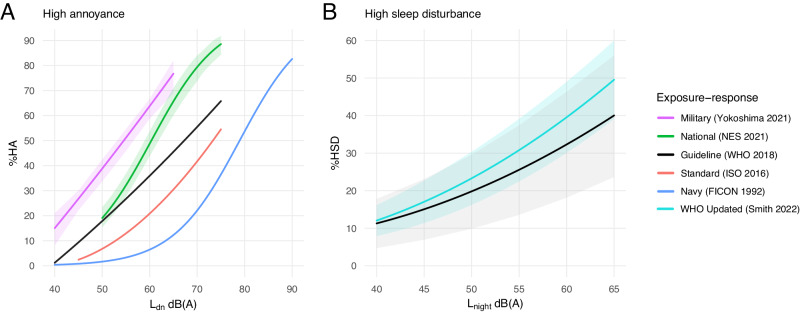
Exposure-response relationships. Functions relating L_dn_ to high annoyance (**A**) and L_night_ to high sleep disturbance (**B**) are illustrated with shaded 95% confidence intervals, where available.

Population health impacts, evaluated according to the number of individuals estimated to experience an adverse health outcome due to noise exposure, were calculated using established exposure-response relationships for annoyance, sleep disturbance, and compromised childhood learning (Fig. 3). These health outcomes were selected because they serve as critical indicators of community health [2–4], they are ubiquitous in noise law (e.g. environmental assessment [30], land-use [27, 47, 48]), and they have published exposure-response relationships that are commonly implemented in domestic and international policy and standards to assess health outcomes from noise [9, 11, 16, 49]. In particular, WHO guidelines identify these outcomes as having sufficiently robust exposure-response relationships to support quantitative health assessment [9]. These outcomes are also the first responses in a stress-mediated chain of physiological effects that can lead to more severe health consequences. Noise exerts effects either directly though objective sound exposure (hearing impairment or sleep disturbance) or indirectly through the subjective emotional and cognitive perception of sound (annoyance) [1, 4, 50]. Both of these pathways elicit neurobiological stress responses that in turn promote cardiovascular risk factors (blood pressure, glucose levels) that can manifest in disease (hypertension, ischemic heart disease) [1, 4, 50, 51] or induce psychological effects that jeopardize mental health (anxiety, depression) [4, 50, 52].

These downstream health outcomes, namely cardiometabolic and psychological effects, were excluded from consideration in the present study because they currently lack generalized exposure-response relationships for public health assessment and are not widely used in domestic and international noise policy and guidelines. While relationships have been quantified for cardiometabolic and psychological effects [52–54], inconclusive empirical support and methodological differences between studies has precluded the development of robust generalized exposure-response relationships [53] and led to the exclusion of these health outcome assessments from WHO guidelines [9, 55, 56]. The chosen outcomes of annoyance, sleep disturbance, and childhood learning serve as proven indicators of community health that can be used to inform policy and prioritize future primary assessments of additional health outcomes from members of the population directly.

While most international noise policies and guidelines rely on L_den_ as the primary cumulative noise metric [9, 11, 49], a majority of US states (including Washington) do not apply a penalty to the evening time period, and instead rely on L_dn_. As such, operational flight profile data from the simulation models were only available in day-night periods, and the following health analyses use L_dn_ in lieu of L_den_. This is expected to result in slightly more conservative estimates than would be expected if L_den_ were available, given that aircraft flight operations were not uncommon during evening hours.

To predict prevalence of high annoyance and high sleep disturbance throughout the population, associated exposure-response functions were used to obtain an estimated percentage of the population impacted from the noise exposure level at the 30 m^2^ spatial grain (raster). Levels exceeding the defined range of a function were capped at the maximum predicted response value, while levels below were assigned a value of zero. The estimated population of each raster was multiplied by this percentage and summed across all units within the study area to estimate the total population subject to each health outcome.

#### Annoyance

Exposure-response curves quantifying the relationship between aircraft noise exposure and human annoyance can differ dramatically by region, community, and type of aircraft and activity. Similarly, curves used in public health policy vary widely between nations. For example, the dose-response curve endorsed by the Federal Interagency Committee on Noise (FICON) [40] remains the current US standard for estimating community response to noise exposure, and is employed by the FAA and Navy. However, the recent comprehensive Neighborhood Environmental Survey (NES) conducted by the FAA found that this standard does not reflect the current US public perception of aviation noise and provided an updated and nationally representative exposure-response curve [12]. Exposure-response curves developed and recommended by the ISO and WHO represent intermediate responses for a given noise exposure level [9, 11].

Although these relationships are commonly applied in the implementation of health risk assessment and noise policy related to commercial and civil aircraft noise, there is evidence that they may underestimate impacts of noise from military aircraft due to the dramatic differences in the frequency and intensity of military aircraft events [12, 19, 20, 22, 57]. For these reasons, we include a unique exposure-response relationship developed by Yokoshima et al., based on a synthesis of individual studies on aircraft noise from US military and Japan Self-Defense Forces [19]. Collectively, these five exposure-response curves were used to assess the range of predicted impacts by relating aircraft noise L_dn_ to the probability of a population being highly annoyed (Fig. 3A).

#### Sleep disturbance

Substantial evidence supports the considerable and consistent effects of aircraft noise on sleep disturbance [9, 58]. These exposure-response relationships are based on survey and experimental assessments that identify aircraft noise as the cause of awakenings from sleep, the process of falling asleep, and/or sleep disturbance. Nighttime noise exposure near military airfields has been found to substantially reduce sleep quality [20, 59]. However, because these studies are highly limited in number, exposure-response curves relating sleep disturbance to military aircraft noise exposure are not available. As such, we employed two published exposure-response curves that relate nighttime aircraft noise L_night_ to the probability of being highly sleep disturbed (Fig. 3B), namely, the guideline curve presented by the WHO and an updated version of this curve by Smith et al. that includes more recent survey data [9, 58]. As previously discussed, these curves are expected to result in conservative estimates of impacted populations.

#### Childhood learning

We investigated the noise exposure levels at geographic centers of public, private, and postsecondary schools within the study area, obtained from the National Center for Education Statistics [60]. Systematic reviews conducted by the WHO and National Academies of Sciences, Engineering, and Medicine found evidence for a negative effect of aircraft noise exposure on reading and oral comprehension, standardized assessment performance, and long-term and short-term memory in children at school [5, 61]. Specifically, WHO guidelines identify an increased risk of impaired reading and oral comprehension at 55 dB L_den_, equating to a 1 month delay in reading age, and an additional 1–2 month delay for each 5 dB increase beyond 55 dB L_den_ [9]. As simulations produced estimates of L_dn_ for the average annual day, assuming 365 days of exposure, we derived this noise level specific risk for each school according to its level of equivalent continuous exposure over a school year duration of 180 days.

#### Hearing impairment

Environmental noise pollution associated with military airfields and military operating areas can occur at levels that can result in both short- and long-term hearing impairment [62–64]. An exposure-response curve directly relating cumulative noise exposure to hearing impairment has not been developed at the population scale. Instead, acute noise exposures that could impact hearing were calculated and compared against action levels for occupational noise according to protocols established by the Occupational Safety and Health Administration and the National Institute for Occupational Safety and Health [65, 66]. Because this analysis requires measurement of continuous sound levels over time as opposed to cumulative metrics, daily noise exposure doses using a 24-h reference duration (representing potential exposure experienced by residents) were calculated for monitoring locations only. Single-event noise levels were also compared against established thresholds for direct physiological impairment [24, 67].

## Results

### Military aircraft noise regime

**Fig. 4:**
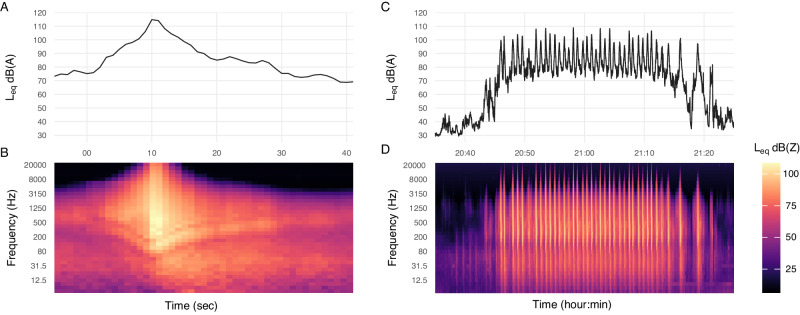
Examples of military aircraft noise events. A single FCLP noise event L_eq_ (**A**) and spectrogram (**B**); an FCLP training session (**C**, **D**). Data recorded at monitoring location 7 on August 10, 2021.

Noise events from military aircraft operations often exhibited a characteristic contour, with a fast onset rising to a maximum peak, followed by a gradual decay (Fig. 4). The magnitude, onset rate, and duration of events varied by operation and monitoring location across the study region.

The loudest aircraft noise event measured at each monitoring location ranged from 85.4–119.8 dB L_max_ and 94.3–122.7 dB L_E_ (Supplementary Table S2). At all monitoring locations on Whidbey Island, as well as off-island locations in Port Townsend (location 11) and the Skagit River (location 2), events were measured in excess of 100 dB L_max_. Noise events during active operations at the MOA location were recorded up to 90 dB L_max_, 95.4 dB L_E_. The single loudest event occurred during a touch-and-go operation near OLF Coupeville (location 15) measuring 119.8 dBA L_max_, 121.1 dBA L_Fmax_, 136.2 dBC L_Cpeak_, 122.7 dBA L_E_, at the time of approximately 23:57.

Spectral energy of aircraft noise occupied an expansive range of the frequency spectrum extending beyond the limits of human audibility. The amount of high frequency noise increased with proximity to aircraft, though substantial amounts of low-frequency energy were present in all noise events, regardless of distance. Energy-averaged Z-weighted levels of one-third octave band spectra from locations within approximately 3 km of an active FCLP session exhibited a broad peak around roughly 300 Hz of 65 to 85 dBZ, and up to 69 dBZ at infrasonic frequencies and 51 dBZ at 20 kHz.

During the four 2020–2021 monitoring periods, Ault Field conducted a wide range of operations, including Olympic MOA departure, arrival, and pattern operations, while the vast majority of operations at OLF Coupeville were FCLPs. Inter-field transit operations were common between both airfields. Ault Field conducted an average of approximately 1134 operations, including 145 FCLPs, per weeklong monitoring period, while OLF Coupeville conducted an average of 760 operations, including 690 FCLPs [33]. A single FCLP counts as two operations, one for takeoff and one for landing. The average weekly number of combat training operations conducted within the Olympic MOA was 66.

Flight operations were concentrated from Monday through Thursday (91.4%), with less activity on Friday (7.1%) and minimal activity on weekends (1.4%). On Friday, Saturday, and Sunday, operations were only conducted from Ault Field. Roughly 70% of operations occurred during daytime hours, 20% during the evening, and 10% at night. Operations were recorded at all hours except 2:00 and 4:00 AM. FCLP sessions at OLF Coupeville took place during the hours of 11:30 to 23:30, ranging in duration from 30 min to 3 h (lasting approximately 1 h on average), with multiple sessions typically occurring on a single day. Sessions have continued past midnight during other recorded monitoring periods [35]. The vast majority of operations within the Olympic MOA occurred during weekdays, with roughly 97% occurring during the day, and 3% at night.

L_den_ increased dramatically on days with substantial flight activity. Compared to weekends (little operation at Ault Field, none at OLF Coupeville), weekdays at monitoring locations within and immediately surrounding Whidbey Island exhibited energy-averaged increases ranging from approximately 8–28 dB L_den_, except for those at Lopez Island (location 3) and Port Townsend (location 11) where differences were negligible. Similarly, L_night_ increased during weekdays, with differences ranging from approximately 3–29 dB. Average hourly L_eq_ increased by up to roughly 35 dB during flight operations, compared to hours when no operations were occurring. As an increase of 10 dB is typically perceived by the human ear as a doubling in loudness, this equates to a roughly 11-fold increase in loudness during hours of operation.

### Simulation model validation

The Navy determined that Noisemap modeling “operates as intended and provides an accurate prediction of noise exposure levels from aircraft operations for use in impact assessments and long-term land use planning” [37]. For each location within the 2020–2021 monitoring period (excluding the Olympic MOA), we compared the estimated L_dn_ from our simulation against results from the Navy’s monitoring study, namely a) the modeled DNL, simulated by Noisemap per-period and then energy-averaged across periods, and b) the real-time measured DNL, calculated from discrete event SEL metrics computed directly from acoustic monitoring data. We found that our simulated results were highly correlated with both the modeled and measured DNL, with Pearson correlation coefficients of 0.99 (*P* < 0.001) and 0.97 (*P* < 0.001), respectively (Supplementary Fig. S1). The mean difference between our simulated L_dn_ and the modeled DNL was 0.72 dB (min 0, max 3), while the mean difference with the measured DNL was 4.25 dB (min 0.9, max 8.2), falling within the accepted range expected by the Navy’s monitoring programs [33]. Simulation of airspace activity within the Olympic MOA yielded a cumulative noise exposure of approximately 35 dB L_dnmr_ throughout the area, approximately 4.8 dB less than the measured energy-average of 39.8 dB DNL during periods of operations across the entire 365-day period.

### Population noise exposure

**Fig. 5:**
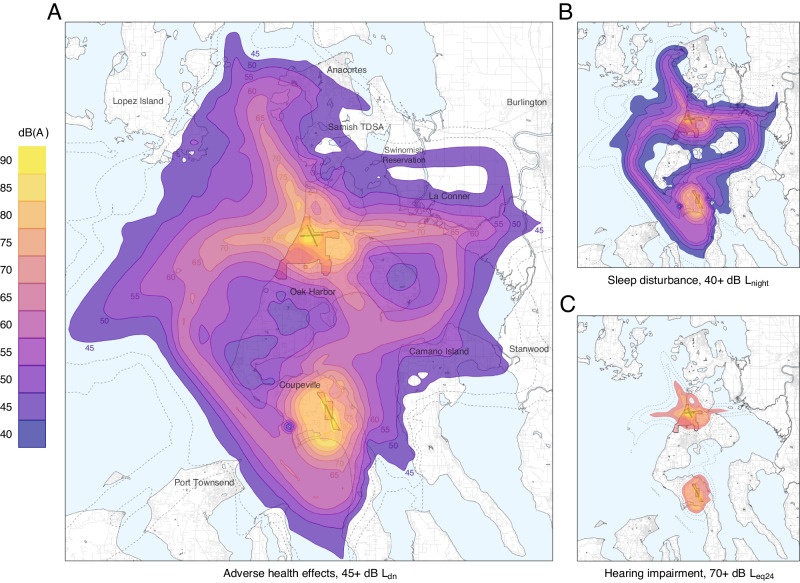
Simulated noise exposure associated with adverse health effects, displayed as contours in 5 dB increments. L_dn_, day-night average sound level, from 45 dB (**A**); L_night_, night average sound level, from 40 dB (**B**); L_eq24_, 24-h equivalent continuous sound level, from 70 dB (**C**). Additional contours below risk thresholds are shown as dotted lines for context.

**Fig. 6:**
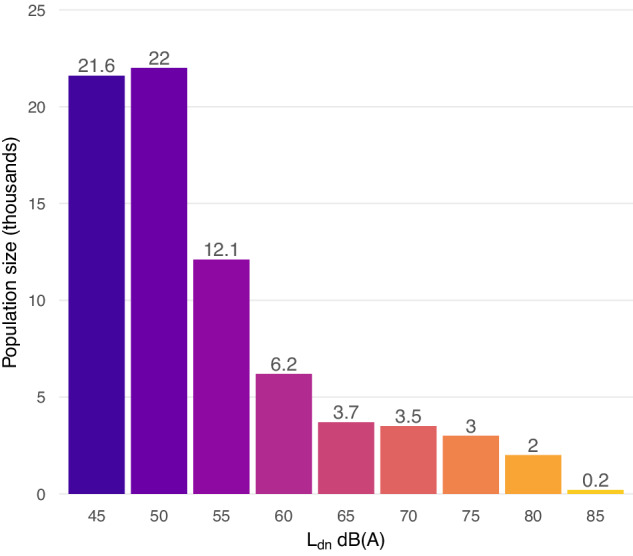
Estimated population at risk of adverse health outcomes. Population size, in thousands, is illustrated per 5 dB L_dn_ of military aircraft noise exposure (lower bound inclusive).

The total area of noise exposure associated with adverse health effects was 1278 km^2^ (427.5 km^2^ not including water) (Fig. 5), with an estimated exposed population of 74,316 people (Fig. 6). Exposure was most severe along flight tracks for airbase arrival, departure, and closed-pattern routines, with L_dn_ reaching beyond 90 dB near landing strips at both Ault Field and OLF Coupeville. Interfacility transit operations also substantially contributed to the spatial extent of noise exposure, exhibiting L_dn_ contours of up to 65 dB.

Simulated operations within the Olympic MOA resulted in a cumulative noise exposure of approximately 35 dB L_dnmr_ throughout the area, with measured individual monthly L_dn_ ranging from approximately 34–47 dB L_dn_. Because the annual average was below the 45 dB L_dn_ threshold, we determined that no health impacts could be estimated for the MOA.

Estimated populations at risk of adverse health outcomes were substantial (Table 1). Island County was the most severely impacted region in terms of total number of exposed people (56,813), amounting to approximately two thirds of the total county population. This included all residents of the cities of Oak Harbor and Coupeville. The Swinomish Reservation was the most severely impacted region in terms of the percentage of community exposure, at roughly 85% of the local reservation population. A total of 74,316 people were exposed to day-night average levels of at least 45 dB L_den_, posing a risk of annoyance [9], while 41,089 were exposed to nighttime average levels of at least 40 dB L_night_, posing a risk of adverse effects on sleep [9]. An estimated 8059 people, all residing within fairly close proximity to Ault Field or OLF Coupeville, were exposed to average noise levels exceeding 70 dBA L_eq24_ that can induce hearing impairment over time [3, 39]. All monitoring locations on Whidbey Island during the same monitoring periods exceeded this level on at least one day, including those lying outside of 70 dBA L_eq24_ contours. A total of 12,449 people were exposed to 65 dB L_dn_ or greater, noise levels incompatible with residential land use according to regulations set by the Navy, FAA, and US Department of Housing and Urban Development, and eligible for community noise mitigation funds [27, 33, 47, 48, 68].

**Table 1:** Summary of population subject to adverse health risks from military aircraft noise exposure.

Region	Population	Exposed	Annoyance	Sleep disturbance	Hearing impairment	Land use
Island County	85,938	56,813 (66.1)	56,813 (66.1)	36,966 (43)	8059 (9.4)	12,449 (14.5)
Skagit County	128,228	17,365 (13.5)	17,365 (13.5)	4089 (3.2)	0	0
Jefferson County	32,590	75 (0.2)	75 (0.2)	34 (0.1)	0	0
San Juan County	17,631	63 (0.4)	63 (0.4)	0	0	0
Samish TDSA	40,853	8194 (20.1)	8194 (20.1)	1925 (4.7)	0	0
Swinomish Reservation	3207	2718 (84.8)	2718 (84.8)	923 (28.8)	0	0
Total	264,387	74,316	74,316	41,089	8059	12,449

“Exposed” refers to the population residing within the 45 dB L_dn_ contour, the WHO threshold for adverse health effects. Percentage of total subpopulation is indicated for each region in parentheses. Note that totals for each outcome do not equal the sum of each individual region, as the population of tribal lands (Samish TDSA and Swinomish Reservation) is included in their coinciding counties.

### Population health impacts

The estimated population health impacts vary according to different published benchmarks (Table 2). According to WHO guidelines, 20,840 people were estimated to be highly annoyed. This estimate ranges from 5873 to 36,916 depending on the exposure-response function used (FICON or Yokoshima, respectively). A total of 5265 people were estimated to be exposed to levels at or beyond the defined range of all annoyance exposure-response functions except FICON (75 dB L_dn_). Between 8315 to 9770 people were estimated to be highly sleep disturbed (WHO versus Smith). Importantly, 4967 people were estimated to be exposed to levels at or beyond the defined range of sleep disturbance exposure-response functions (65 dB L_night_).

**Table 2:** Summary of estimated population subject to high annoyance and high sleep disturbance from military aircraft noise exposure.

		Highly annoyed					Highly sleep disturbed	
Region	Population	FICON	ISO	WHO	NES	Yokoshima	WHO	Smith
Island County	85,938	5604 (6.5)	9765 (11.4)	18,110 (21.1)	21,776 (25.3)	30,689 (35.7)	7778 (9.1)	9179 (10.7)
Skagit County	128,228	267 (0.2)	771 (0.6)	2714 (2.1)	1559 (1.2)	6185 (4.8)	533 (0.4)	587 (0.5)
Jefferson County	32,590	1 (<0.1)	2 (<0.1)	9 (<0.1)	0	23 (0.1)	4 (<0.1)	4 (<0.1)
San Juan County	17,631	1 (<0.1)	1 (<0.1)	7 (<0.1)	0	19 (0.1)	0	0
Samish TDSA	40,853	125 (0.3)	361 (0.9)	1276 (3.1)	734 (1.8)	2912 (7.1)	254 (0.6)	280 (0.7)
Swinomish Reservation	3207	47 (1.5)	140 (4.4)	457 (14.3)	320 (10)	1013 (31.6)	120 (3.7)	132 (4.1)
Total	264,387	5873	10,539	20,840	23,335	36,916	8315	9770

Percentage of total subpopulation is indicated for each region in parentheses. Note that totals for each outcome do not equal the sum of each individual region, as the population of tribal lands (Samish TDSA and Swinomish Reservation) is included in their coinciding counties.

Several monitoring locations within roughly 3 km of OLF Coupeville surpassed the recommended exposure limit of the National Institute for Occupational Safety and Health (≥85 dB time-weighted average). Days with multiple FCLP sessions occasionally surpassed exposure thresholds for the FAA Hearing Conservation Program, which would require providing hearing protection and testing for employees [69]. Multiple noise events at a residence near OLF Coupeville (location 15) reached short-term intensities that may cause direct, acute mechanical damage to the inner ear [24, 67].

Six schools were exposed to noise levels associated with increased risk of reduced reading and oral comprehension (≥55 dB L_dn_, Supplementary Table S3) [9], with the quantitative risk of a 1–2 month delay in learning per 5 dB increase. The most severely impacted schools (Coupeville Middle School, Coupeville High School, and Crescent Harbor Elementary) had an estimated exposure of 60–63 dB L_dn_, suggesting the risk of a 2–3 month delay in learning for students. An additional 12 schools were exposed to noise levels within 5 dB of the 55 dB L_dn_ threshold. Columbia College, a postsecondary school near Ault Field, experienced exposure of 70 L_dn_; impacts were not able to be estimated, however, given that the average student is older than the ages for which this relationship is defined.

Particularly loud individual aircraft noise events occurred during school hours at schools in proximity of flight tracks. At the field monitoring location nearest Crescent Harbor Elementary (location 1, distance of approximately 1 km), events surpassed 103 dB L_max_ and 113 dB L_E_. Considering a standard 15–25 dB reduction to approximate indoor levels, noise events of such magnitude are known to interfere with student and teacher conversation and comprehension [70–72].

### Simulation of alternative noise regimes

**Fig. 7:**
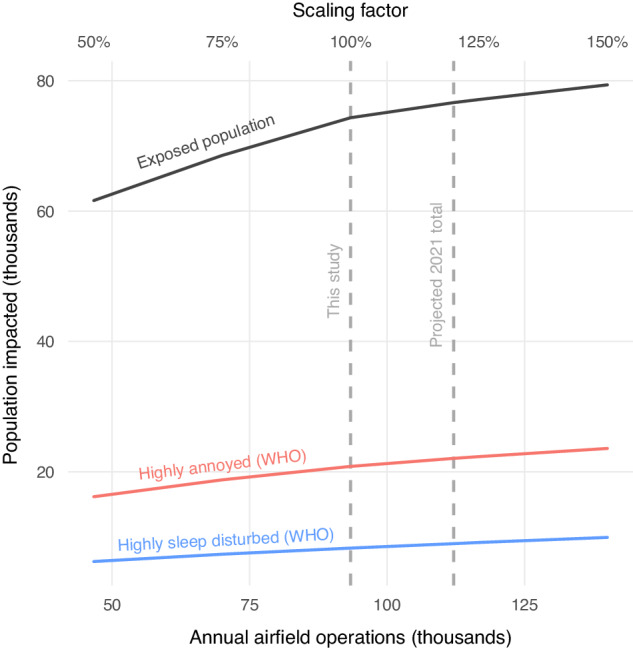
Changes in estimated health impacts from varying volume of simulated aircraft operations. The equivalent annual number of operations for the four monitoring periods and the total number of operations projected for 2021 from the Navy environmental impact statement are both shown as vertical dashed lines.

Simulation of alternative noise regimes revealed a positive relationship between aircraft operations and the estimated population impacted by all health outcomes across a wide range of total operations (Fig. 7). This relationship is nonlinear, as it is dependent on both the spatial distribution of the population and the range of levels for which thresholds and exposure-response relationships are defined. A 50% increase in annual operations yielded a 6.8% increase in the total exposed population, while a 50% decrease in operations yielded a 37% decrease in the exposed population. A 50% increase in operations increased cumulative noise levels within the Olympic MOA to no more than 36.4 dB L_dnmr_.

## Discussion

Pervasive noise pollution from military aviation in western Washington exposes communities to an array of risks and impacts to human health and well-being. A majority of countries, including the United States, lack regulations that limit environmental noise pollution. This supports the needs for approaches, such as the one presented here, that allow for quantifying population health risks and impacts relative to established exposure-response relationships and regulatory guidelines from other noise-focused policies (e.g., occupational noise, land-use compatibility for civilian airports). Through the estimation of potential health burdens and projected increases and decreases in aircraft operations, this approach can inform impacted communities, highlight points of interest and areas that should be prioritized for alleviation and noise mitigation strategies, and better inform aircraft operational design, urban planning, and the development of improved policies to protect the public from the adverse human burden of noise exposure.

Our results suggest that noise exposure due to military aircraft activities within the study region poses a substantial risk and impact to public health. A majority of the resident population of Island County (66%) were exposed to noise levels associated with adverse health effects. Using WHO guidelines, 21% were estimated to be highly annoyed and 9% to be highly sleep disturbed. The Swinomish Indian Tribal Community of the Swinomish Reservation was extremely vulnerable to health risks, with nearly 85% of residents being exposed. The greatest impacts were predicted for populations residing near airfields and flight tracks, though the effects permeate broadly across the landscape. Our predictive modeling of health outcomes focuses on annoyance and sleep disturbance as critical indicators of community health for which sufficient research exists to employ exposure-response relationships. Ultimately, these outcomes are the first responses in a chain of physiological effects that can result in more severe health impacts. Stress responses can be triggered as a downstream consequence of sleep disturbance or the emotional and cognitive perception of sound (annoyance), promoting cardiovascular and psychological risk factors [4, 52]. Mounting evidence from recent laboratory and longitudinal studies point to these underlying physiological and neurobiological mechanisms as pathways through which noise increases the risk of, or leads to, the onset of disease [4, 50–52]. This strongly suggests that noise exposure should be considered as an environmental risk factor for the development of cardiometabolic and psychological disease and disorder [50]. While our study did not include a primary assessment of health outcomes from members of the population directly, the scale and severity of the indicators presented here imply considerable and diverse impacts to public health that warrant further investigation.

The estimated extent of population impacts varies widely depending on the standard or exposure-response relationship that is employed. For example, use of the FAA’s newly revised NES exposure-response curve for high annoyance, as opposed to the current FICON standard, increased population health impacts by almost 4-fold. Health impacts can also vary across individuals, and exposure-response relationships are influenced by the life experience and culture of the populations from which they are derived [19]. This wide range in estimated impacts suggests that there is uncertainty associated with current methods for health assessment, and conventional thresholds governing permissible community exposure, such as the 65 dB L_dn_ contour used by the Navy, FAA, and US Department of Housing and Urban Development for land-use compatibility, may fail to protect populations from the adverse effects of noise exposure they were designed to. Despite this, the exposure-response relationships used in this study are currently employed in health assessments at an international scale, meaning that estimated public health risks and associated costs to society will vary widely. We suggest that variation in population health impact estimates starkly illustrates the need to reduce uncertainties in our understanding of how noise exposure results in human health outcomes and identify levels of permissible exposure informed by the best available science.

Critical gaps remain in our ability to assess health outcomes from military aviation noise specifically, and environmental noise pollution in general. First, an important finding of this study was that a substantial portion of the population was exposed to noise levels at or beyond the defined range of exposure-response relationships. This indicates that these levels of exposure are unprecedented in community noise analyses. Although there are few circumstances outside of close proximity to military airfields where such exposure is likely to be routine, it remains that the expected community response and health impacts from aircraft noise exposure at such extreme levels is unknown. Second, our simulation also produced contours for noise levels below those commonly presented in conventional assessments, but still proven to elicit adverse health effects. For example, domestic assessments often limit contours to 65 dB L_dn_ and above [33], while the Environmental Noise Directive requires that assessments report population estimates exposed to 55 dB L_den_ and above [49], despite evidence that adverse human health effects can be experienced at 45 dB L_dn_ [9]. When considering an entire population, even comparatively low levels of exposure can yield substantial societal effects. As such, we urge future health assessments to consider the entire range of noise exposure known to be harmful to human health.

Perhaps the most significant knowledge gap involves the difference in estimated impacts between the exposure-response curves for noise from military versus civil aircraft [19, 25]. This difference is dramatic and warrants further study to understand the role of acoustic (both single-event and cumulative metrics) and non-acoustic indicators in future community health assessments of noise from military aviation. For example, military aircraft noise within the study region exhibited substantial low-frequency energy. The conventional use of A frequency weighting underestimates the contribution of this energy to noise measurements and has been criticized as inadequate to quantify such low-frequency noise events [73, 74]. Low-frequency noise propagates farther than higher frequency noise, and therefore A-weighted contours may underestimate the true spatial extent of noise exposure. Additionally, low-frequency noise is not easily attenuated by physical barriers such as walls and windows and can resonate with building structures and the human body, creating secondary effects of rattling and vibration [73, 75, 76]. This minimizes the efficacy of sound insulation and can promote feelings of helplessness in residents [73]. While the specific health impacts of low-frequency noise exposure remain understudied, evidence suggests that it can elicit a substantially more rapid increase in perceived loudness, annoyance, and sleep disturbance, even at low sound pressure levels [3, 73, 75, 76]. The WHO and ISO suggest that guidelines for permissible exposure to low-frequency noise should be lowered and rating procedures for the assessment of such noise should be modified [11]. However, there remains little consensus on what modifications are appropriate due to a dearth of research relating low-frequency noise exposure to health impacts. Future improvements to noise simulation tools such as Noisemap should include the ability to calculate C-weighted levels to better quantify exposure to low-frequency noise.

Military aircraft activity within the study region also exhibited a more sporadic noise regime than that associated with common commercial or civil aviation, which may contribute to more severe health impacts than are predicted by cumulative acoustic metrics such as L_dn_ [22]. Individual events can have significant implications for health outcomes that cumulative metrics fail to account for. This suggests that communities outside of the 45 dB L_dn_ contour may still be subject to noise exposure that is associated with adverse health effects. For example, although most residents of Port Townsend, Camano Island, Lopez Island, and Forks (a city within the Olympic MOA) reside outside the area that poses a relevant risk of annoyance according to the WHO, these communities have reported high numbers of noise complaints [29]. This may be exacerbated in areas within the Olympic MOA in particular, such as Forks, due to the intermittent nature of noise events and comparatively reduced presence of other environmental noise [18]. However, current research linking single-event indicators to long-term population health impacts remains tentative, and as such these metrics are not widely employed in assessment guidelines [9].

Simulations of alternative noise regimes suggest that population health impacts can be slightly reduced, but definitely not avoided, by decreasing the volume of aircraft operations. While increased operations are associated with more severe health impacts, the spatial extent of noise exposure (and the mere presence of health risks) is driven in particular by the flight pattern of active operations. Therefore, reducing population exposure and subsequent health impacts may best be achieved by strategically discontinuing or altering the flight paths of operations that result in the most egregious impacts, such as FCLP operations, as well as reducing the volume. Changing the timing of operations to avoid sensitive periods, such as school and nighttime hours, may also substantially reduce the risk of associated health impacts. Further opportunities may exist in the creation of subsidy programs for sound insulation or even the purchase of homes in high exposure areas, such as those for commercial airports in the US [27, 71] or military airfields abroad [20, 25]. However, it should be noted that although sound insulation has been shown to reduce indoor exposure to traffic and commercial aircraft noise [77, 78], prior research suggests that it may be ineffective in reducing the high intensity and low-frequency noise associated with military aircraft [20].

Finally, the spatial extent of noise exposure presented in this study, and thus the magnitude of predicted health risks and impacts, is likely underestimated due to limitations of available data. The scaling of operations occurring within the four weeklong monitoring periods to the entire year amounted to 83% of the volume of total operations suggested by the Navy for all of 2021 [30]. Compared to our simulation, modeled yearly average L_dn_ values presented in the Navy environmental impact statement are louder at most monitoring locations, some by up to 10.5 dB [37], and the area of the 65 dB L_dn_ contour is approximately 1.23 times as large (not including water or the NASWI complex) [30]. This suggests that human health impacts from the true annual noise regime are likely even more severe than those shown here.

The approach presented here improves on existing methods of assessing the population health risk of noise by increasing transparency and reproducibility. Although acute and chronic noise pollution is an issue for many communities worldwide, noise and operations from NASWI have been closely scrutinized for many years. This has led to unique monitoring datasets and a rare legal action that critically examined, and ultimately rejected, the environmental impact statement process and procedures as implemented by the Department of Defense [79]. Other recent successful lawsuits also underscore the inadequacy of current environmental assessment practices and policies related to noise [80], and the burden on communities to organize and self-advocate for change [81]. Our approach offers several benefits and improvements for noise impact assessment. The first is that we rely on published exposure-response relationships to transparently and systematically estimate impacts for multiple health outcomes. These relationships can be expanded or updated as new information becomes available (e.g., exposure-response curves for cardiovascular or psychological impacts). A second improvement lies in the integration of dasymetric population density maps which provide a more accurate estimation of exposed populations, particularly across different types of non-urban areas such as those prevalent in the study region. This is important as noise pollution is not an exclusively urban issue, extending well outside city centers. Finally, this workflow can be used to project the expected magnitude and geography of population health risk resulting from proposed changes in activity, including both increases and decreases in flight operations related to new policy and mitigation strategies (e.g., land use changes, sound insulation programs). We believe that this approach can be employed by both noise producers and affected communities as a basis for common dialogs that extend beyond noise exposure to discuss human health impacts and potential solutions.

Military aviation operations data are typically not available to the public, and noise monitoring around airfields is rare. This study purposefully leveraged datasets that arose from unique policy instruments [33] and robust monitoring funded by federal agencies [21] and community organizations [35, 36]. We believe this approach offers a roadmap that communities elsewhere can use to effectively plan and implement rigorous noise monitoring and systematically collect operational data. For example, the datasets that facilitated this assessment have common features, including the use of class 1 sound level meters to collect an array of SPL measurements and a robust means to identify individual events automatically or through simultaneous observation for comparison with operations data. The workflow presented makes more accessible the conversion of these raw acoustic data into meaningful metrics for communication. Using this example, community-led initiatives could advocate for government funding to support noise monitoring studies or conduct their own investigations with volunteers. The ongoing evolution of inexpensive monitoring equipment and advances in acoustic analysis increase this likelihood, with strong potential for application of AI-based automated detection of noise events from long-term passive monitoring [82]. Such datasets collected through community science could provide useful validation of noise modeling and exposure [83, 84], although overcoming sporadicity must be considered [85]. This may offer a much-needed mechanism to organize and advocate for mitigation and could go a long way to alleviating the powerlessness that communities commonly experience related to noise pollution [86].

By using a modular analytical framework, this approach can be extended to diverse noise sources well beyond the purview of military aircraft, including any context where the spatial extent of noise exposure can be modeled. This offers a promising avenue to bridge monitoring gaps for other environmental noise sources that are poorly documented and monitored yet still affect communities worldwide. The framework can also be readily updated or customized with other health thresholds and exposure-response relationships to serve future assessments in other contexts. The insights obtained through this approach may help better inform efforts in mitigating community noise exposure and developing policy governing noise legislation and land use. We strongly encourage future public health assessments of environmental noise pollution to leverage such a workflow in an effort to obtain a comprehensive understanding of the magnitude of health implications associated with noise exposure at the population scale with the best available science.

## Supplementary information

Supplementary Information

Table-S1

## Data Availability

Code for the methods and workflow routines can be found at the open-source repository github.com/giojacuzzi/noise-pollution-pop-health-naswi [87]. Noisemap software is available from the US Department of Defense Community and Environmental Noise Primer resources at dodnoise.org/primer_resources. BaseOps version 7.368 was used as the graphical user interface for Noisemap simulation data entry and management in concert with a) Omega10 and Omega11 to calculate sound over distance for aircraft flight operations, ground maintenance, and run-up operations; b) NMap 7.3 and MRNMap to calculate noise exposure values on the ground; c) NMPlot 4.974 to convert calculated noise exposure values to noise contour plots. Data from the 2020–2021 Navy monitoring periods are available from the Naval Facilities Engineering Systems Command Aircraft Sound Monitoring database [34], including acoustic monitoring data, flight operations data, and noise modeling data. Acoustic monitoring data from the 2015 National Park Service Night Skies and Sounds Division report and 2016 and 2019 JGL Acoustics, Inc. reports are available upon reasonable request. Impervious surface data used in the dasymetric population density mapping are available from the National Land Cover Database [46]. School geographic locations are available from the National Center for Education Statistics [60].
