# Supplementary material for: Population health implications of exposure to pervasive military aircraft noise pollution
Source: J Expo Sci Environ Epidemiol. 2024 May 9;35(1):91–103. doi: 10.1038/s41370-024-00670-1 (PMC11876064; doi:10.1038/s41370-024-00670-1)

Supplementary Information: Population health implications of exposure to pervasive military aircraft noise pollution

Giordano Jacuzzi, Lauren M. Kuehne, Anne Harvey, Christine Hurley, Robert Wilbur, Edmund Seto, Julian D. Olden

Supplementary tables, figures, and associated legends are provided below.

Table S1: Catalog of acoustic field monitoring data.

(See supplementary file Table S1)

Table S2: Maximum single noise event metrics, ordered by L_{max} dB(A), per monitoring location.

Location	L_{max}	L_{Fmax}	L_{Cpeak}	L_E
15	119.8	121.1	136.2	122.7
7	115.1	115.8	130.3	119.5
19	115.0	117.6	–	117.2
13	115.0	116.6	130.5	117.0
8	114.8	116.5	123.7	118.8
9	113.8	115.0	124.1	118.6
4	110.2	112.0	124.2	114.1
18	109.6	110.7	126.4	115.6
5	109.1	111.6	124.5	116.1
16	108.2	111.4	128.2	114.0
6	108.0	108.7	123	114.3
17	107.6	110.1	127.1	113.7
14	104.3	106.7	–	110.6
10	104.2	105.8	122.1	111.9
2	104.1	105.8	122.4	110.5
1	103.5	103.9	120.6	112.2
11	101.1	106.9	124.3	112.3
3	90.7	93.2	107.5	97.5
12	90.0	92.6	101.4	95.4
20	85.4	–	–	94.3

Table S3: Schools with adverse noise exposure at risk of delay in childhood learning.

School	***Exposure (L_{dn})***	***Delay (Months)***
Coupeville High School	63	2-3
Coupeville Middle School	63	2-3
Crescent Harbor Elementary	60	2-3
Coupeville Elementary School	59	1
Island County Corrections Facility Ed Program	57	1
Olympic View Elementary	56	1

Fig. S1: Comparison of simulated L_{dn} with Navy modeled and measured DNL for monitoring locations 1-11.

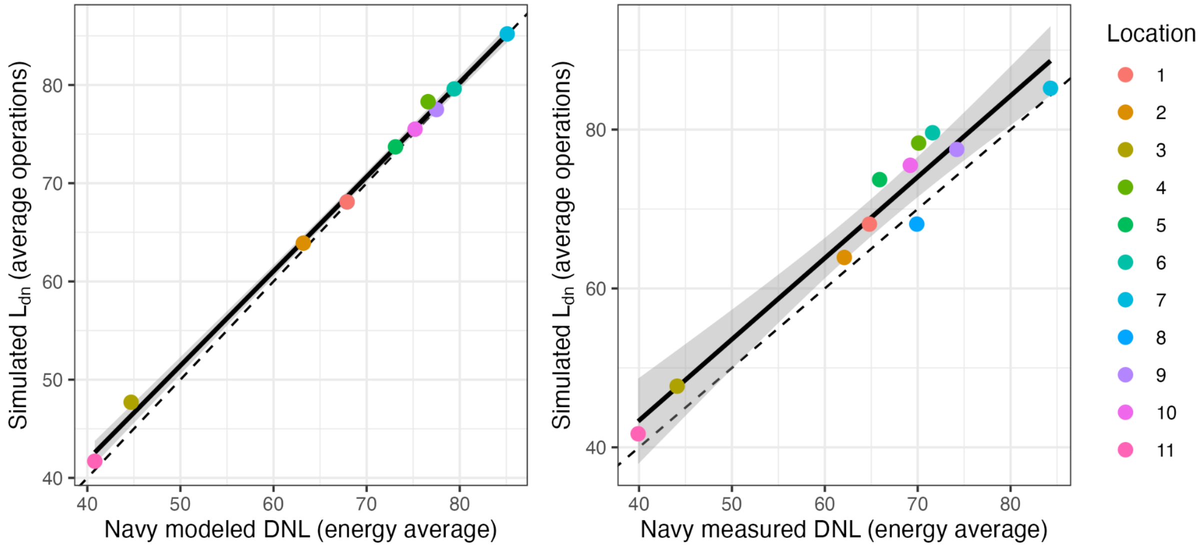

Supplement: Supplementary file 1 — Supplementary Information [file 41370_2024_670_MOESM1_ESM.pdf]
